# Detection of Oxidation Products of 5-Methyl-2′-Deoxycytidine in Arabidopsis DNA

**DOI:** 10.1371/journal.pone.0084620

**Published:** 2013-12-31

**Authors:** Shuo Liu, Thomas L. Dunwell, Gerd P. Pfeifer, Jim M. Dunwell, Ihsan Ullah, Yinsheng Wang

**Affiliations:** 1 Environmental Toxicology Graduate Program, University of California Riverside, Riverside, California, United States of America; 2 Beckman Research Institute, City of Hope Medical Centre, Duarte, California, United States of America; 3 School of Agriculture, Policy and Development, University of Reading, Reading, Berkshire, United Kingdom; 4 Agricultural Biotechnology Research Institute, Faisalabad, Pakistan; 5 Department of Chemistry, University of California Riverside, Riverside, California, United States of America; Chinese Academy of Science, China

## Abstract

Epigenetic regulations play important roles in plant development and adaptation to environmental stress. Recent studies from mammalian systems have demonstrated the involvement of ten-eleven translocation (Tet) family of dioxygenases in the generation of a series of oxidized derivatives of 5-methylcytosine (5-mC) in mammalian DNA. In addition, these oxidized 5-mC nucleobases have important roles in epigenetic remodeling and aberrant levels of 5-hydroxymethyl-2′-deoxycytidine (5-HmdC) were found to be associated with different types of human cancers. However, there is a lack of evidence supporting the presence of these modified bases in plant DNA. Here we reported the use of a reversed-phase HPLC coupled with tandem mass spectrometry method and stable isotope-labeled standards for assessing the levels of the oxidized 5-mC nucleosides along with two other oxidatively induced DNA modifications in genomic DNA of Arabidopsis. These included 5-HmdC, 5-formyl-2′-deoxycytidine (5-FodC), 5-carboxyl-2′-deoxycytidine (5-CadC), 5-hydroxymethyl-2′-deoxyuridine (5-HmdU), and the (5′*S*) diastereomer of 8,5′-cyclo-2′-deoxyguanosine (*S*-cdG). We found that, in Arabidopsis DNA, the levels of 5-HmdC, 5-FodC, and 5-CadC are approximately 0.8 modifications per 10^6^ nucleosides, with the frequency of 5-HmdC (per 5-mdC) being comparable to that of 5-HmdU (per thymidine). The relatively low levels of the 5-mdC oxidation products suggest that they arise likely from reactive oxygen species present in cells, which is in line with the lack of homologous Tet-family dioxygenase enzymes in Arabidopsis.

## Introduction

DNA methylation at the C5 position of cytosine is a conserved epigenetic mark for transcriptional gene silencing in diverse organisms [1]. While the levels of 5-methylcytosine (5-mC) are relatively low in human genomes (∼4% of total cytosine), 5-mC is abundantly present in plant genomes (5–25% depending on the species) [2]. In addition to the primary methylation at CG sites, cytosine in plants can also be methylated in CHG and, less frequently, in CHH sequences (‘H’ represents A, C or T) [3]. The plant methylation patterns are established by different methyltransferase activities. *De novo* domains rearranged methyltransferases (DRMs) transfer methyl groups to completely unmethylated duplex DNA in all sequence contexts, and chromomethylase 3 (CMT3) can convert cytosine to 5-mC at non-CG sites [4]. The CG methylation is propagated during mitotic cell divisions by a group of maintenance methyltransferases (MET1 in Arabidopsis) [5].

Another mechanism for the dynamic regulation of the methylation status of genes is to passively and/or actively remove 5-mC from DNA. Passive demethylation occurs when cells fail to maintain the methylation during DNA replication. In plants, a subfamily of helix-hairpin-helix-Gly/Pro/Asp (HhH-GPD) DNA glycosylases have been identified as demethylases involved in active cytosine DNA demethylation [6]. These bifunctional glycosylases remove the 5-mC base and then cleave the DNA backbone at the resulting abasic site. Subsequent action by base excision repair (BER) machinery results in the replacement of 5-mC with an unmethylated cytosine [7]. Previous studies demonstrated the biological role of the glycosylase-mediated demethylation of DNA in Arabidopsis. Loss-of-function mutations in *Demeter* (*DME*), a 5-mC DNA glycosylase gene in Arabidopsis, lead to impaired endosperm and embryo development, and eventually in seed abortion [8]. Hypermethylation of cytosine occurred in genomes of plants that lack members of the DNA glycosylase demethylase family, e.g. repressor of silencing 1 (ROS1), DME-like 2 (DML2), and DME-like 3 (DML3) [9].

Mammals appear to lack the activity of glycosylases that can excise 5-mC specifically. However, active DNA demethylation may also be achieved in mammals through a BER pathway by DNA glycosylases, though it requires oxidation of 5-mC as the first step [10]. In this vein, 5-mC is converted into 5-hydroxymethylcytosine (5-HmC), 5-formylcytosine (5-FoC), and 5-carboxylcytosine (5-CaC) by the ten-eleven translocation (Tet) family of DNA dioxygenases through iterative oxidation [11], [12]. 5-FoC and 5-CaC, but not 5-HmC, at CG site can be readily removed by thymine DNA glycosylase and replaced with unmethylated cytosine by BER proteins [13], [14]. This differential reactivity toward thymine DNA glycosylase may account for the recent observations that 5-FoC and 5-CaC are much less abundant than 5-HmC in mammalian genomes [12], [15].

The discovery of Tet-induced oxidation of 5-mC in mammals raised questions about the possible presence of consecutive oxidations of 5-mC in plants. The mammalian TET proteins responsible for these oxidative modifications contain a catalytic domain that is typical of Fe(II)- and 2-oxoglutarate (2OG)-dependent dioxygenases, members of the cupin superfamily [16]. By using computational analysis, Iyer et al. [17] reported several distinct families of Fe(II)- and 2OG-dependent dioxygenases that are likely to be involved in oxidation of 5-mC in mammals and other early branching eukaryotes such as fungi and algae. In contrast, enzymes of this family have not been identified in multicellular plants [17], [18]. The prediction of lack of this cytosine modifying enzymatic activity in plants was strengthened by the fact that, despite some preliminary results reporting the presence of 5-HmC [19], [20], there is yet no definitive evidence supporting the presence of oxidation products of 5-mC in plants.

As it was considered that this conclusion was based on the absence of evidence rather than the evidence of absence, we set out to assess the levels of these modified bases in Arabidopsis DNA using a liquid chromatography coupled with tandem mass spectrometry (LC-MS/MS/MS) method. In this context, we recently reported the application of LC-MS/MS/MS, along with the isotope-dilution technique, for sensitive and accurate quantification of 5-hydroxymethyl-2′-deoxycytidine (5-HmdC), 5-formyl-2′-deoxycytidine (5-FodC), 5-carboxyl-2′-deoxycytidine (5-CadC), and 5-hydroxymethyl-2′-deoxyuridine (5-HmdU) in mammalian cells and tissues [15]. Herein, we measured these four modified nucleosides along with the (5′*S*) diastereomer of 8,5′-cyclo-2′-deoxyguanosine (*S*-cdG), a reliable biomarker for endogenously induced oxidative DNA damage [21], in Arabidopsis DNA. Our results demonstrated the presence of these modified nucleosides in Arabidopsis. Their relatively low levels in genomic DNA, however, suggest that, in contrast to the observations made for mammals, they are not likely to be formed from enzyme-mediated oxidation reactions.

## Materials and Methods

**Figure 1:**
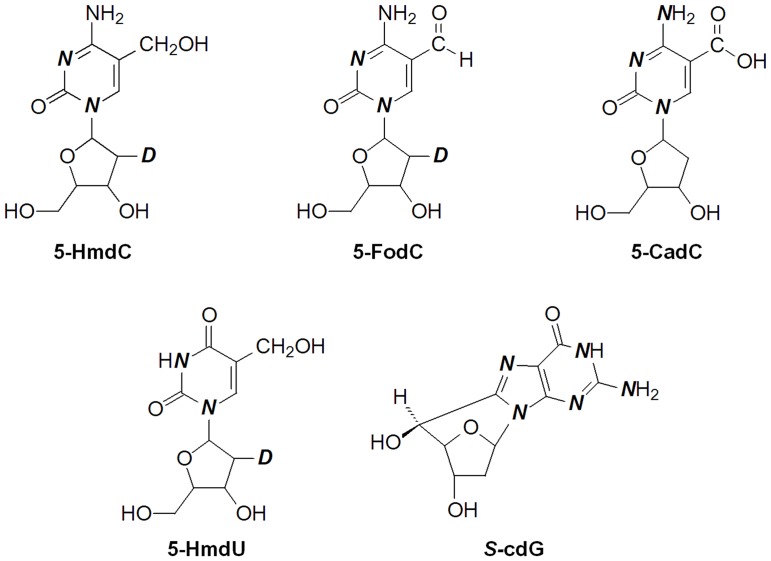
Chemical structures of the modified nucleosides measured in this study. The “N” in bold italic font represents ^15^N, and the site of deuterium atom incorporation is indicated with a “D”.

Genomic DNA was isolated from leaves of four individual plants of *Arabidopsis thaliana* Columbia (Col-0) using a DNeasy Plant Maxi Kit (Qiagen, UK) according to the manufacturer’s instructions. Nuclease P1 and phosphodiesterases 1 and 2 were from Sigma-Aldrich (St. Louis, MO). Alkaline phosphatase was obtained from New England Biolabs (Ipswich, MA), and erythro-9-(2-hydroxy-3-nonyl)adenine (EHNA) hydrochloride was from Tocris Bioscience (Ellisville, MO). [1,3-^15^N_2_, 2′-D]-5-HmdC, [1,3-^15^N_2_-2′-D]-5-FodC, [4-*amino*-1,3-^15^N_3_]-5-CadC, [1,3-^15^N_2_-2′-D]-5-HmdU, and [1,3,7,9-^15^N_4_-(2-*amino*-^15^N)]-*S*-cdG were synthesized previously [15], [22], [23], [24]. The chemical structures of these modified nucleosides are shown in Figure 1.

Plant DNA (30 µg) was first digested by nuclease P1 (0.1 U/µg DNA) and phosphodiesterase 2 (0.00025 U/µg DNA) in a solution containing 30 mM sodium acetate (pH 5.6), 1.0 mM zinc acetate, and 1 mM EHNA, which is an inhibitor for adenine deaminase. After incubation at 37°C for 48 h, Tris-HCl (pH 8.9) was added to the solution until its final concentration reached 50 mM and the samples were further treated with alkaline phosphatase (0.05 U/µg DNA) and phosphodiesterase 1 (0.0005 U/µg DNA) at 37°C for 2 h. To the mixture were then added 90 fmol of [1,3-^15^N_2_, 2′-D]-5-HmdC, 30 fmol of [1,3-^15^N_2_-2′-D]-5-FodC, 75 fmol of [4-*amino*-1,3-^15^N_3_]-5-CadC, 2 pmol of [1,3-^15^N_2_-2′-D]-5-HmdU, and 128 fmol of [1,3,7,9-^15^N_4_-(2-*amino*-^15^N)]-*S*-cdG. The enzymes were subsequently removed from the digestion mixture by chloroform extraction. The aqueous layer was subjected to off-line HPLC for the enrichment of these five target nucleosides.

The enrichment was carried out on a Beckman HPLC system with pump module 125 and a UV detector (module 126). A 4.6×250 mm Aeris Widepore C18 column (3.6 µm in particle size, Phenomenex, Torrance, CA) was used. An isocratic elution at a flow rate of 0.8 mL/min was employed with a solution of 10 mM ammonium formate (pH 8.5) as mobile phase. The HPLC fractions for 5-HmdC, 5-FodC, 5-CadC, 5-HmdU and *S*-cdG were pooled, dried, redissolved in water, and then injected for LC-MS/MS/MS analysis.

The LC-MS/MS/MS experiments were conducted using a 0.5×250 mm Zorbax SB-C18 column (5 µm in particle size, Agilent Technologies, Santa Clara, CA) and an Agilent 1200 capillary HPLC pump. A solution of 0.1% (v/v) formic acid in water (solution A) and a solution of 0.1% (v/v) formic acid in methanol (solution B) were used as mobile phases. A gradient of 5 min 0–20% B and 25 min 20–70% B was employed for the separation of the modified nucleosides. The flow rate was 6.0 µL/min. The effluent from the LC column was directed to an LTQ linear ion-trap mass spectrometer (Thermo Fisher Scientific, San Jose, CA). The temperatures of the ion transport tube were maintained 275 and 300°C in the positive- and negative-ion modes, respectively. The electrospray, capillary and tube lens voltages were 5 kV, 4 V and 25 V, respectively, in the positive-ion mode, and 4.5 kV, −12 V and −92 V, respectively, in the negative-ion mode. The sheath gas flow rate was 15 arbitrary units, and no auxiliary gas was used. The mass spectrometer was set up to acquire the MS^3^ spectra for fragmentations of the [M+H]^+^ ions of 5-HmdC, 5-FodC, 5-CadC, and *S*-cdG, and the [M-H]^−^ ion of 5-HmdU following previously described methods [15], [24].

## Results

**Figure 2:**
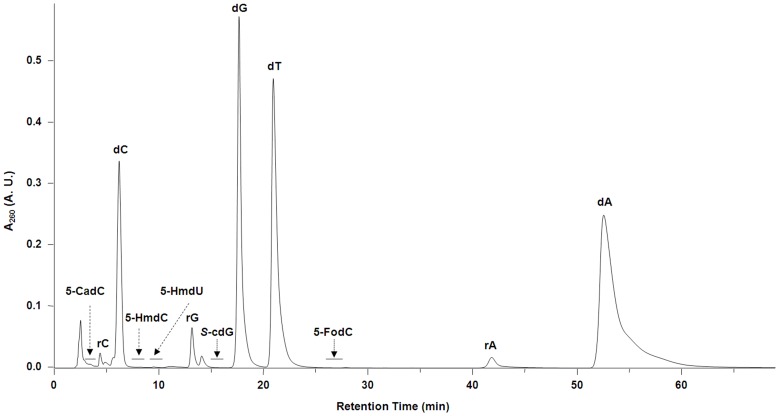
Representative HPLC trace for the enrichment of 5-HmdC, 5-FodC, 5-CadC, 5-HmdU, and *S-*cdG from the enzymatic digestion mixture of genomic DNA isolated from Arabidopsis.

**Figure 3:**
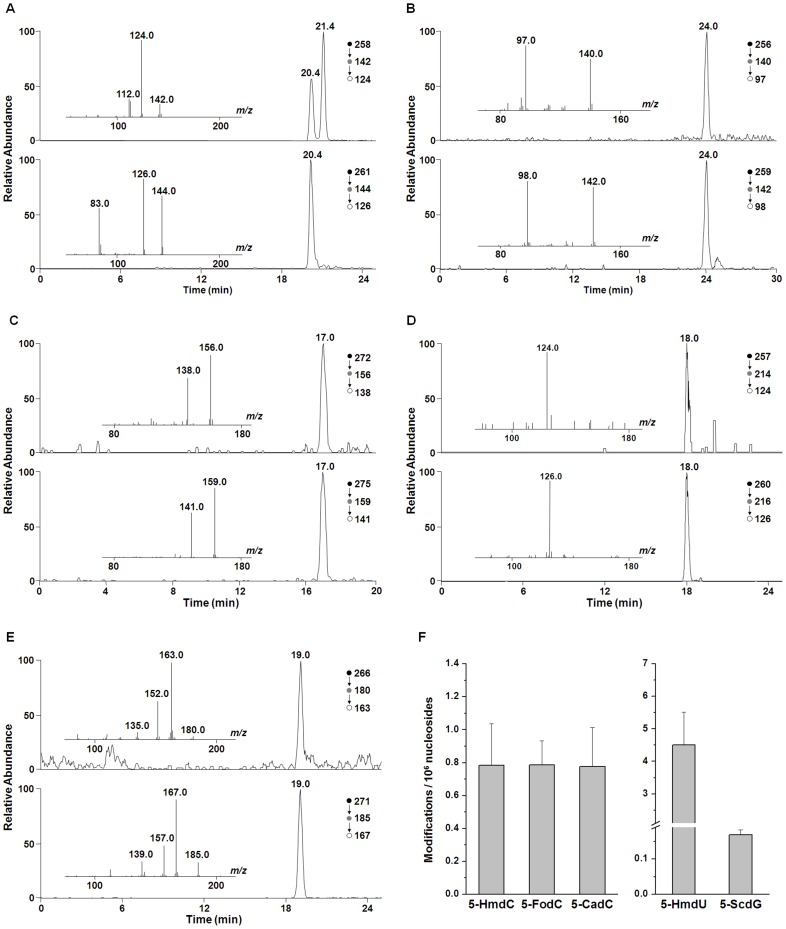
Representative LC-MS/MS/MS results for the quantification of 5-HmdC (A), 5-FodC (B), 5-CadC (C), 5-HmdU (D), *S-*cdG (E), and quantification results (F) in Arabidopsis DNA. Shown in panels A-E are the selected-ion chromatograms for monitoring the indicated transitions for the analytes (top trace) and the isotope-labeled standards (bottom trace), and the insets give the MS/MS/MS results for the analytes and internal standards.

As a first step toward exploring whether the oxidation of 5-mC plays a role in epigenetic regulation in plants, we employed our recently developed LC-MS/MS/MS coupled with the stable isotope-dilution technique [15], [24] to measure the levels of 5-HmdC, 5-FodC, and 5-CadC in genomic DNA from Arabidopsis leaves. For comparison, we also quantified 5-HmdU and *S*-cdG in the same DNA samples. In this regard, we utilized HPLC to enrich these modified nucleosides prior to LC-MS^3^ analysis, as described previously [15]. As shown in Figure 2, the targeted analytes were well resolved from each other and from the canonical ribonucleosides and 2′-deoxyribonucleosides. The identities and quantities of the aforementioned nucleosides were established from LC-MS/MS/MS measurements. The selected-ion chromatograms (SICs) and MS^3^ spectra for unlabeled and labeled analytes are displayed in Figure 3. The fragmentation behaviors of the protonated ions of 5-HmdC, 5-FodC, 5-CadC, and *S*-cdG and the deprotonated ion of 5-HmdU as well as the calibration curves for each analyte were previously described [15], [24]. Briefly, collisional activation of protonated ions of the three modified 5-mdC nucleosides gave rise to cleavages of the N-glycosidic linkage and facile elimination of a 2-deoxyribose moiety. Further fragmentation of protonated ions of the nucleobase portion yielded one major fragment ion (*m/z* 142, 140, and 138 for 5-HmdC, 5-FodC, and 5-CadC, respectively) emanating from the neutral losses of H_2_O for 5-HmdC and 5-CadC, and HNCO for 5-FodC. On the other hand, collisional activation of the [M+H]^+^ ion of *S*-cdG leads to the facile cleavages of the N-glycosidic linkage and the bond between C4′ and C5′ of 2-deoxyribose to give the ion of *m/z* 180 in MS/MS. Further collisional activation of the *m/z*-180 ion results in the losses of NH_3_, CO, and both to generate the ions of *m/z* 163, 152, and 135 in MS/MS/MS. As reported previously, 5-HmdU could be detected with better sensitivity in the negative- than positive-ion mode [25]. Upon collisional activation, the [M-H]^ −^ ion of 5-HmdU loses an HNCO from the modified nucleobase moiety to produce the most abundant ion of *m/z* 214 in MS/MS, further activation of which leads to elimination of part of the 2-deoxyribose component to give the ion of *m/z* 124 in MS^3^ [15]. Corresponding fragments were observed in the MS^3^ of the stable isotope-labeled standards (Figure 3). The identical elution times in SICs and similar MS^3^ spectra for the analytes and their stable isotope-labeled standards confirmed the identities of the modified nucleosides and allowed for their reliable quantification.

With the use of this method, we successfully detected 5-HmdC, 5-FodC, 5-CadC, 5-HmdU, and *S*-cdG in four independent genomic DNA preparations from Arabidopsis leaves (Table 1). Our results revealed that 5-HmdC, 5-FodC, and 5-CadC were present at very similar levels in Arabidopsis genomic DNA (0.79, 0.79, and 0.78 per 10^6^ nucleosides, respectively, Figure 3). In addition, we found that the levels of 5-HmdU and *S*-cdG were approximately 4.5 and 0.17 modifications per 10^6^ nucleosides, respectively (Figure 3).

**Table 1:** Frequencies of 5-HmdC, 5-FodC, 5-CadC, 5-HmdU and *S*-cdG (in the unit of modifications per 10^6^ nucleosides) in Arabidopsis genomic DNA.

Nucleosides	Numbers* (n = 4)
5-mdC/100 dG	9.63±0.92
dT/100 dN	19.2±0.18
5-HmdC/10^6^ dN	0.79±0.25
5-FodC/10^6^ dN	0.79±0.15
5-CadC/10^6^ dN	0.78±0.24
5-HmdU/10^6^ dN	4.50±1.01
*S-*cdG/10^6^ dN	0.17±0.01

The data represent mean ± standard deviation.

## Discussion

The combination of LC-MS^3^ with the stable-isotope dilution technique provides a sensitive and accurate method for the measurement of oxidation products of 5-mdC, together with 5-HmdU and *S*-cdG, in Arabidopsis tissues. To our knowledge, this is the first rigorous quantification of all three 5-mdC modification products in a flowering plant. Since the recent discoveries of Tet-mediated oxidation of 5-mdC to 5-HmdC, 5-FodC, and 5-CadC, many biophysical and biochemical techniques have been employed for their detection, including LC-MS analysis [26], [27], [28], [29], thin layer chromatography [30], chemical derivatization followed by sequencing analysis [31], [32], single molecule detection [33], antibody-based dot-blot analysis [11], [20], [34], etc. The development of these methods has provided valuable insights regarding the roles of these 5-mdC oxidation products in processes such as active DNA demethylation in mammals. By using a dot-blot assay, Yao *et al.* [20] first reported the observation of low levels of 5-HmC in Arabidopsis leaves and flowers. However, a dot-blot assay does not offer an accurate quantification of the modified nucleobase. The relatively low levels of oxidized derivatives of 5-mC in Arabidopsis require more sensitive methods for their reliable detection. With the use of our recently developed LC-MS^3^ coupled with isotope-dilution method, we were able to detect these modified bases in Arabidopsis DNA. In contrast to the high levels of 5-HmdC found in mammals (∼200–1600 modifications per 10^6^ nucleosides), which were about 100 to 1000-fold higher than those of 5-FodC and 5-CadC, our results revealed that the Arabidopsis genome contains similar levels (approximately 0.8 modifications per 10^6^ nucleosides, Figure 3) of 5-HmdC, 5-FodC, and 5-CadC.

While 5-HmC, 5-FoC, and 5-CaC are considered as products from Fe(II)- and 2OG-dependent dioxygenase-mediated oxidation, they could also arise from endogenous reactive oxygen specie (ROS). Thus, we also measured 5-HmdU and *S*-cdG to estimate the contribution of endogenous ROS to the formation of 5-HmdC, 5-FodC, and 5-CadC. The results showed that the levels of the three 5-mdC modification products are ∼0.8 modifications per 10^6^ nucleosides, whereas the level of 5-HmdU is approximately 5.7-fold higher than that of 5-HmdC. Considering that there are approximately 6.5 times more thymidine than 5-mdC nucleosides in Arabidopsis, the frequency of occurrence of 5-HmdC (on per mdC basis) is only 1.1 time as high as that of 5-HmdU (on per dT basis). Thus, at least part of 5-mdC modification products arises from ROS present in cells or from artificial oxidation of 5-mdC in DNA during various steps of sample preparation. The latter, however, may make a smaller contribution according to the results for *S*-cdG measurements. The 8,5′-cyclopurine-2′-deoxynucleosides are considered as robust biomarkers for oxidative stress. We previously measured the levels of *S*-cdG and 5-HmdC in mouse skin tissues, which were at the levels of 0.35 and 200 modifications per 10^6^ nucleosides, respectively [15], [35]. In comparison with the results obtained in this study, the difference is huge in the level of 5-HmdC but not that of *S*-cdG between mammalian and Arabidopsis tissues. Given that the level of 5-HmdC in a plant genome is ∼2–3 orders of magnitude lower than that in mammalian tissues while 5-FodC and 5-CadC are present at similar levels [12], [15], [29], it is unlikely that in plants these oxidized bases serve as intermediates in active DNA demethylation. This finding is in keeping with the fact that to date no putative homologues of Fe(II)- and 2OG-dependent dioxygenase enzymes responsible for 5-mdC oxidation have been unambiguously identified in plants [17].

In summary, we found that Arabidopsis genomic DNA contains detectable levels of oxidation products of 5-mC. Our quantification results suggest that the modified bases are most likely induced by ROS, though we cannot exclude the possibility that intermediates of iterative oxidation of 5-mdC are present at a small number of specific genomic loci. Further studies would be needed to determine whether 5-HmC, 5-FodC, and 5-CadC are located at specific loci in Arabidopsis DNA, but this approach would be challenging given the low levels of these modified bases that we measured.
